# Evaluation of dexmedetomidine as an adjuvant to low-concentration lidocaine/ropivacaine mixtures in ultrasound-guided axillary brachial plexus block

**DOI:** 10.1186/s12871-025-03221-9

**Published:** 2025-07-17

**Authors:** Qi Wang, Lu Feng

**Affiliations:** Department of Anesthesiology, The Second Affiliated Hospital of Harbin Medical University, Harbin, Heilongjiang Province 150001 China

**Keywords:** Brachial plexus block, Dexmedetomidine, Analgesic, Sedative

## Abstract

**Background:**

Dexmedetomidine (DEX) can be used with local anesthetics (LAs) to enhance the efficiency of a peripheral nerve block. However, there have been few studies on the combination of DEX and two different LAs for a brachial plexus block (BPB). The effects of adding DEX to low concentrations of lidocaine (LIDO) mixed with ropivacaine (ROP) on block onset, duration of anesthesia, and efficacy of analgesia in ultrasound-guided axillary (UGA) BPB were investigated.

**Methods:**

The study protocol was approved by the Ethics Committee of the Second Affiliated Hospital of Harbin Medical University (ChiCTR-IPR-16007742, January 12, 2016), China. Seventy-five patients designated as American Society of Anesthesiologists Physical Status Classification System I or II and scheduled for forearm or hand surgery were assigned to three groups: (1) R group (*n* = 25), 0.25% ROP (30 mL) with 0.9% NaCl (3 mL); (2) RL group (*n* = 25), 0.25% ROP (15 ml) and 0.5% LIDO (15 mL) with 0.9% NaCl (3 mL); and (3) RLD group (*n* = 25), 0.25% ROP (15 ml) and 0.5% LIDO (15 mL) with DEX (0.75 µg/kg) (3 mL). Data on hemodynamic alterations, the bi-spectral index score (BIS), occurrence and timing of sensory and motor blocks, duration of analgesia, and requirement for rescue analgesia > 48 h were collected.

**Results:**

The timings of the onsets of sensory and motor blocks were considerably reduced in the RL and RLD groups relative to the R group (*p* < 0.0001), with no substantial variation between the RL and RLD groups (*p* > 0.05). Compared with the R and RL groups, the analgesic and block periods in the RLD group were markedly increased (*p* < 0.0001). The requirement for flurbiprofen rescue intervention was markedly reduced in the RLD group relative to the R and RL groups (*p* < 0.0001). The BIS was markedly lower in the RLD group, between 20 and 60 min (*p* < 0.05).

**Conclusion:**

The combination of ROP and LIDO led to a reduction in the onset time in UGA BPB. The addition of DEX to ROP/LIDO prolonged the duration of sensory and motor blocks. DEX also resulted in an extension of the analgesia time and provided significant sedation.

**Supplementary Information:**

The online version contains supplementary material available at 10.1186/s12871-025-03221-9.

## Background

Brachial plexus block (BPB) is an inexpensive and widely applied technique that provides efficient post-operative analgesia during hand and forearm surgeries. However, the prolonged time required to obtain a complete block after administering local anesthetics (LAs) limits their applicability in procedures requiring rapid execution. This constraint restricts its suitability for enhanced recovery after surgery protocols and can lead to patient anxiety [1].

The efficacy and safety of using a mixture of LAs for peripheral nerve blocks (PNBs) remains unclear and has been debated for many years. The mixing of LAs can alter the pH, thereby influencing the onset time. When the pH approaches the physiological level (7.4), the proportion of non-ionized (lipid-soluble) LAs increase, enhancing their ability to penetrate the nerve membrane, reducing the onset time. Some studies reported that combining two LAs does not substantially affect the onset time or duration of analgesia in BPB [2, 3]. Conversely, other studies [4] have demonstrated that mixtures of two LAs can achieve satisfactory anesthesia and analgesia in BPB. Furthermore, combined LAs have been shown to have the advantage of a more rapid onset with a simultaneously reduced block duration than single long-acting LAs [5–7].

The incorporation of various adjuvants, including clonidine [8, 9], dexmedetomidine (DEX) [10–14], dexamethasone [13–16], tramadol [17, 18], and magnesium sulfate [13, 19, 20] into PNBs has been shown to enhance block potency by reducing the onset time, extending the analgesic time, decreasing the required doses of LAs, and reducing their toxic effects. Moreover, DEX, a highly specific α_2_-adrenergic receptor agonist, has been extensively studied for its efficacy in prolonging and intensifying anesthesia. Numerous trials have shown the safety and effectiveness of DEX when administered *via* epidural [21, 22], intrathecal [23, 24], and peripheral routes [25, 26]. In addition to its analgesic effects mediated *via* spinal cord receptors, DEX induces a sedative effect by interacting with locus ceruleus receptors [27]. Previous studies have revealed that perineural administration of DEX promotes a state of clarity and alleviates patient anxiety [28].

To date, studies have indicated the usefulness of combining DEX with individual long-lasting LAs, such as bupivacaine [29], levobupivacaine [10, 30], or ropivacaine (ROP) [31–33], for BPB. However, studies reporting the use of DEX with two LAs for upper extremity surgery are limited. This study aimed to examine whether a mixture of ROP and lidocaine (LIDO), relative to ROP alone, reduces onset time and whether the addition of DEX to this mixture further accelerates onset, prolongs block duration, and extends analgesia in ultrasound-guided axillary (UGA) BPB.

## Materials and methods

### Study design

**Fig. 1:**
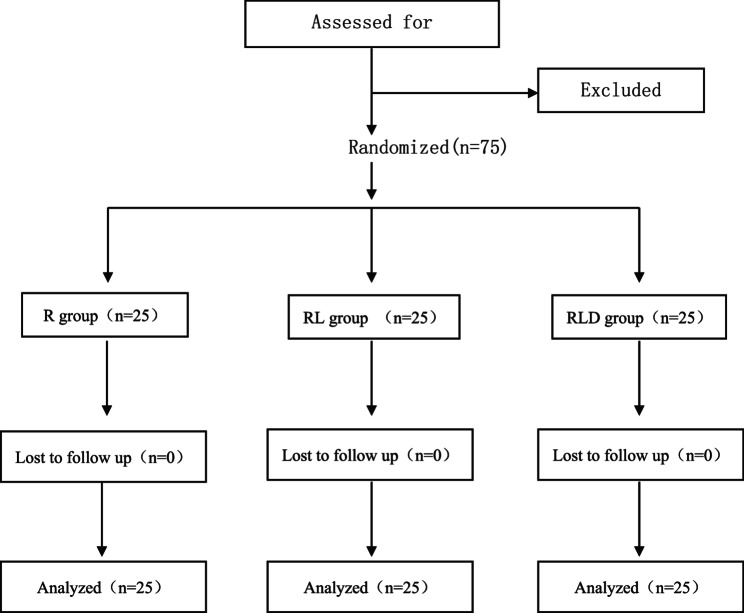
Flow diagram

The study adhered to the Consolidated Standards of Reporting Trials guidelines. Seventy-five patients with American Society of Anesthesiologists Physical Status Classification System Grades I and II participated in this prospective, double-blind, randomized controlled trial (fig 1). The participants (18 to 60 years) were scheduled to undergo hand and forearm surgeries using UGA BPB. Exclusion criteria included patients with known allergies to the study medications, central nervous system disorders, peripheral neuropathy, infection at the puncture site, a history of chronic liver, kidney, heart, or lung diseases, individuals with disruption of coagulation, opioid or alcohol dependency, severe pain, morbid obesity, and pregnancy or lactation.

Patients were randomly assigned to three groups, and the randomization sequence was generated by an independent statistician using a computer-generated random number table with a 1:1:1 allocation ratio. After enrollment, group assignments were obtained using a centralized randomization system (REDCap) to ensure concealment of allocation. Interventions were administered by an anesthesiologist who was not involved in assessing the outcomes, using sealed opaque envelopes containing group assignments; all participants received 33 mL of the respective study solutions:

- R group(*n* = 25), 30 mL of 0.25% ROP (75 mg) + 0.9% NaCl (3 mL).
- RL group (*n* = 25), 15 mL of 0.25% ROP (37.5 mg) and 15 mL of 0.5% LIDO (75 mg) + 0.9% NaCl (3 mL).
- RLD group, (*n* = 25 15 mL of 0.25% ROP (37.5 mg) and 15 mL of 0.5% LIDO (75 mg) + DEX (0.75 µg/kg) (3 mL).

### Drug preparation

ROP was diluted from 1 to 0.25%, LIDO was diluted from 2 to 0.5%, and DEX was diluted to 100 µg/mL. The required volume of DEX was calculated based on patient body weight, precisely drawn using a 1 mL insulin syringe (BD Ultra-Fine™, accuracy: 0.01 mL), and then added to normal saline to achieve a final volume of 3 mL.

### Observation

Lactated Ringer’s solution (5 mL/kg/h) was injected into a lower-extremity peripheral vein via an 18-gauge catheter. A facemask was used for oxygen administration (3 L/min), with no accompanying sedatives. Non-invasive monitoring of systolic arterial pressure (SAP), heart rate (HR), peripheral oxygen saturation (SpO_2_), respiratory rate (RR), diastolic arterial pressure (DAP), and bi-spectral index score (BIS) was performed. An anesthesiologist who was blinded to the agents being administered performed the UGA BPB. The patients were positioned supine with upper-arm abduction of 90° and elbow flexion of 110°. The injection site was prepared and disinfected, and ultrasonography (14 MHz, Terason 2000, USA) was used to identify the veins, axillary arteries, and nerves (ulnar, medial, and radial).

Under guidance, a 22-gauge needle was inserted close to the nerve. Following multiple aspirations to minimize the risk of intravascular injection, each nerve was examined and encased in the LA solution (11 mL) under investigation.

### Primary outcome measures

Blocks were evaluated at 30 s until a complete block was confirmed because the onset time was remarkably rapid. Post-surgery, the block was examined at 30-min intervals until recovery of function. Sensory blocks were evaluated using a three-point pinprick assessment (0 = normal, 1 = dull, and 2 = no sensation), while motor blocks were assessed using a three-point scale (0 = normal function, 1 = only able to move fingers, and 2 = without motor function) [34, 35]. The initiation and duration of sensory and motor blocks and the duration of analgesia were documented. The time of onset was defined as the interval between completion of the injection and loss of pain perception in the three nerves (score 2). Sensory blocks represent the time between sensation loss and return to a normal level (score 0). Motor block onset was defined as the interval between completion of the injection and the ability to move a finger (score 1), whereas the duration of block was defined as the time from onset to the overall restoration of function in the forearm and hand (score 0). The duration of analgesia was defined as the interval between administration of local anesthetics and the first post-operative request for analgesics. Flurbiprofen (50 mg) was administered intravenously as a single dose to patients who reported post-operative pain, and the number of rescue analgesic doses required within 48 h post-operatively was noted.

### Secondary outcome measures

All parameters SAP, HR, DAP, and SpO_2_, were measured at the initial level and 5, 10, 15, 30, 45, and 60 min, post-administration of LA, while BIS was recorded at the initial level and 5, 10, 15, 20, 25, 30, 45, and 60 min, post-injection. Hypotension and bradycardia were defined as a reduction of ≥ 20% from baseline values, while hypertension was defined as an increase in mean arterial pressure of ≥ 20% from baseline. An SpO_2_ level of < 90% indicated respiratory suppression. The duration of surgery and type of surgery were documented. The presence of other adverse effects, including dry mouth, nausea, dizziness, and vomiting were also documented. The anesthesiologists who monitored the patients during the post-operative period and noted any complications were blinded to the group assignments.

The anesthesiologist responsible for evaluating the blocks and the patients were unaware of the contents of the administered material. Patients who did not achieve a complete block within 30 min of injection were not included in the study. For patients who had pain during the procedure, intravenous fentanyl (1 µg/kg) with midazolasm (0.02 mg/kg) were administered; this was documented as a failure and the participants were not included in the trial.

### Statistical analysis

Duration of analgesia was designated as the initial outcome variable. To detect a 30% increase in this variable with 0.9 power and 0.05 threshold value, a sample of 19 participants per group was calculated using SAS 9.13. Statistical analyses were performed by a blinded statistician using SAS 9.13. All demographic and time-related data were examined via repeated measures ANOVA. Both BIS and hemodynamic values were also analyzed using repeated measures ANOVA and single-effect analysis. The chi-square (x²) test assessed sex disparity between groups. The significance threshold was set at *p* < 0.05.

## Results

A suitable block and follow-up data was attained in all the 75 participants. The demographic traits, duration of surgery, and type of surgery were not substantially different; (*p* > 0.05) (Table 1).

The time of onset for both sensory and motor blocks were remarkably shorter in RL (7.95 ± 2.29 min and 12.85 ± 3.88 min, respectively) relative to R (12.89 ± 2.48 min; 20.53 ± 3.44 min), and was reduced in RLD (7.26 ± 2.40 min; 12.66 ± 3.27 min), without substantial disparity between both groups (*p* > 0.05). However, the timings of both sensory and motor blocks in RLD were markedly prolonged (671.06 ± 45.67 min; 624.82 ± 114.50 min) in contrast to both R (548.11 ± 26.05 min; 422.23 ± 39.33 min) and RL (539.64 ± 29.87 min; 430.75 ± 41.87 min) (*p* < 0.05). Furthermore, the duration of analgesia was extended in RLD (778.90.±68.28 min) relative to both R (496.73 ± 57.45 min) and RL groups (508.19 ± 64.94 min) (*p* < 0.0001). The frequency of flurbiprofen administration was considerably lower in the RLD group (1.97 ± 0.45) in contrast to R (4.55 ± 0.85) and RL groups (4.55 ± 0.89) (*p* < 0.0001; Table 2).

**Table 1:** Participant characteristics and surgical parameters

Group	R (*n* = 25)	RD (*n* = 25)	RDL (*n* = 25)	*P*_1_-value	*P*_2_-value	*P*_3_-value	*P*
Age	41.92 ± 9.40	44.52 ± 10.31	45.16 ± 8.14	0.996	0.683	1	0.440
Height	166.72 ± 7.40	168.44 ± 7.44	169.87 ± 10.56	1	0.597	1	0.435
Weight	65.50 ± 11.91	62.88 ± 12.19	63.23 ± 11.67	1	1	1	0.702
Sex (F/M)	12/13	9/16	10/15	1	1	1	0.681
Type of surgery							
Internal fixation of hand fractures	10	9	9	1	1	1	0.945
Tendon repair of the hand	7	8	7	1	1	1	0.931
Soft tissue mass resection of the hand and forearm	8	8	9	1	1	1	0.942
Duration of Surgery(min)	64.13 ± 19.76	70.41 ± 34.68	67.19 ± 28.56	1	1	1	0.736

The groups were comparable (*P*>0.05)

*F* Female, *M* Male, *SD* Standard deviation

Values are mean ± SD. P_1_-value: group RL compared with group R; P_2_-value: group RLD compared with group R; P_3_-value: group RLD compared with group RL

Group R: 30 mL of ropivacaine +3 mL of normal saline; Group RD: 30 mL of lidocaine/ropivacaine + 3mL of normal saline; Group RLD: 30 mL of lidocaine/ropivacaine + 3mL of DEX (0.75 µg/kg)

**Table 2:** Time of onset, length of sensory and motor block, duration of analgesia, and adverse events

Group	R (*n* = 25)	RL (*n* = 25)	RLD (*n* = 25)	*P*_1_-value	*P*_2_-value	*P*_3_-value	*P*
Sensory block onset time (min)	12.89 ± 2.48	7.95 ± 2.29 *	7.26 ± 2.40 *	<0.001	<0.001	0.531	<0.001
Motor block onset time (min)	20.53 ± 3.44	12.85 ± 3.88 *	12.66 ± 3.27 *	<0.001	<0.001	1	<0.001
Sensory block length (min)	548.11 ± 26.05	539.64 ± 29.87	671.06 ± 45.67 ▲*	1	<0.001	<0.001	<0.001
Motor block length (min)	422.23 ± 39.33	430.75 ± 41.87	624.82 ± 114.50 ▲*	1	<0.001	<0.001	<0.001
Length of analgesia (min)	496.73 ± 57.45	508.19 ± 64.94	778.90 ± 68.28▲*	1	<0.001	<0.001	<0.001
Frequency of flurbiprofen administration in 48 h	4.55 ± 0.85	4.55 ± 0.89	1.97 ± 0.49 ▲*	1	<0.001	<0.001	<0.001
Adverse events	None	None	None				

Data represent means ± SD

Flurbiprofen was given in single 50 mg doses IV. Group R: 30 mL of ropivacaine + 3 mL of normal saline; Group RD: 30 mL of lidocaine/ropivacaine + 3 mL of normal saline; Group RLD: 30 mL of lidocaine/ropivacaine + 3 mL of DEX (0.75 µg/kg)

P_1_-value: group RL compared with group R; P_2_-value: group RLD compared with group R; P_3_-value: group RLD compared with group RL

Versus group R, *p* < 0.05*

Versus group RL, *p* < 0.05 ▲

**Fig. 2:**
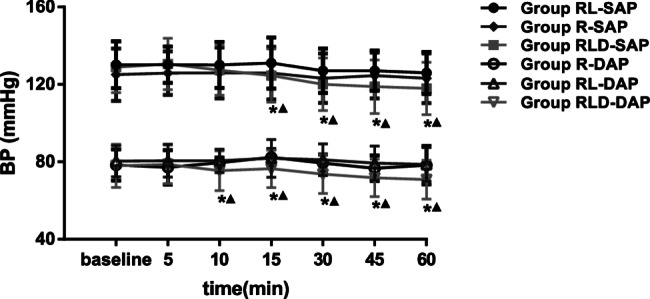
Systolic arterial pressure and diastolic arterial pressure for the two groups. *means time points of statistically significant difference compared with groupR. (*P* <0.05) ▲ means time points of statistically significant differences compared with groupRL. (*P* <0.05) SAP=systolic arterial pressure; DAP=diastolic arterial pressure; BP=blood pressure. Group R:3ml normal saline + 30ml lidocaine; Group RL: 3ml normal saline + 30ml idocaine/ropivacaine; Group D: 3ml DEX (0.75μg kg-1) + 30 ml lidocaine/ropivacaine

**Fig. 3:**
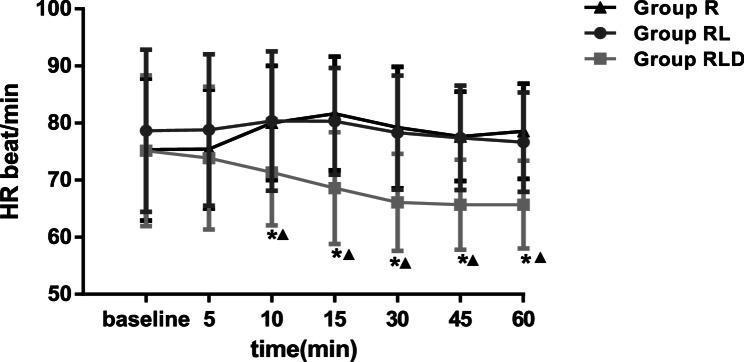
Heart rate for the three groups. *means time points of statistically significant difference compared with groupR. (*P* <0.05) ▲ means time points of statistically significant difference compared with groupRL. (*P* <0.05) SAP=systolic arterial pressure; DAP=diastolic arterial pressure; BP=Blood pressure. Group R:3ml normal saline + 30ml lidocaine; Group RL: 3ml normal saline + 30ml idocaine/ropivacaine; Group D:3ml DEX (0.75μg kg-1) +30ml lidocaine/ropivacaine

**Fig. 4:**
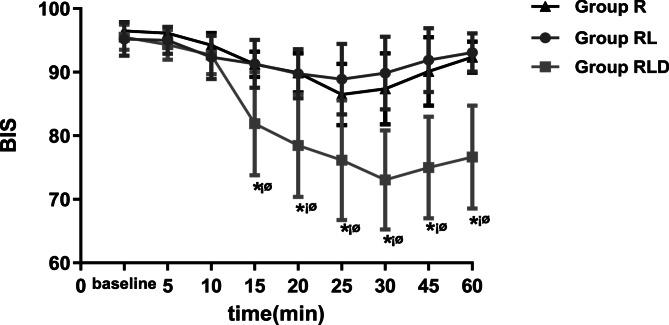
BIS values for the groups. *means time points of statistically significant difference compared with groupR. (*P* <0.05) ¡ømeans time points of statistically significant difference compared with groupRL. (*P* <0.05) SAP=systolic arterial pressure; DAP=diastolic arterial pressure; BP=blood pressure. Group R: 3ml normal saline + 30ml lidocaine; Group RL: 3ml normal saline + 30ml idocaine/ropivacaine; Group D; 3ml DEX (0.75μg¡ kg-1) + 30 ml lidocaine/ropivacaine

Figures 2 and 3 depict SAP, DAP, and HR levels, respectively. Starting at 15 min, the SAP level was substantially reduced in the RLD group relative to the R and RL groups. The HR and DAP in the RLD group were substantially reduced at 10 min compared to those in the other groups. BIS was markedly lower in the RLD group from 20 to 60 min (Fig. 4).

No adverse events were documented in any participant and none required administration of supplementary analgesics (Table 2).

## Discussion

This study found that the combination of ROP and LIDO resulted in a substantially more rapid onset of both sensory and motor blocks than for ROP alone in UGA BPB, without a considerable reduction in block duration. The addition of 0.75 µg/kg DEX to the perineural solution failed to produce a reduction in the onset time, but it prolonged both the timings of sensory and motor blocks, as well as the analgesic condition, in contrast to LAs alone (in the R and RL groups). However, DEX facilitated appropriate sedation and enhanced post-operative analgesia, without inducing hypertension or bradycardia.

LIDO and ROP are weakly acidic (pH approximately 5.5) [36, 37], and their mixture does not significantly alter the pH or proportion of the non-ionized form. The small amount (less than 1 mL of 100 µg/mL) of DEX (also weakly acidic) used has almost no effect on the pH of the mixed local anesthetic. The faster onset observed in the RL group compared to the R group may result from lidocaine’s rapid onset properties and synergistic effects between lidocaine and ropivacaine.

In their meta-analysis, Lin et al. found that combining short- and long-acting local anesthetics modestly reduced the time to surgical block [38], consistent with our findings. However, unlike their study, we observed no associated decrease in sensory/motor block duration in the RL group compared to the R group. We did not find that the vasodilatory properties of LIDO compromised the duration of block using ROP. There is a positive correlation between the potency of the long-acting anesthetic in the mixture and sensory/analgesic duration.

In contrast to the current results, some clinical studies have reported that DEX reduced the onset period relative to LAs alone [30, 32]. It was also predicted that several factors may account for the discrepancy. First, as previously stated, differences in anesthesia techniques and LA administration may have resulted in variations in the distribution of the anesthetic within the plexus sheath, thereby influencing the concentration and effects of the additives [8]. Second, some studies [29, 30, 39, 40] used higher doses of DEX, which may have had a more pronounced effect on onset time. Third, in the current study, the combination of LIDO and ROP already provided a relatively rapid onset time in contrast to R (12.89 ± 2.48 min; 20.53 ± 3.44 min). As a result, the addition of DEX did not lead to a further significant reduction in onset time.

Searches of the literature suggest that the sensory occurrence time of 7.26 ± 2.40 min in the RLD group represents the shortest time ever documented for BPB, at the time this manuscript was prepared. These data reinforce the rationale for a synergistic dose of the two types of LA for BPB. In this study the beginning of motor block in RLD was 12.66 ± 3.27 min, greater than that described in a recent study [41]. This outcome is predicted to be attributable to the use of lower concentrations of ROP (0.25%) and LIDO (0.5%) in the present study, which were adequate to provide sufficient anesthesia but with a relatively diminished effect on motor function. Lower doses were administered to reduce the risk of complications of prolonged motor block. Furthermore, this study found that a few patients retained the ability to move their fingers throughout the surgical procedure, which benefitted them. This observation also justifies the rationale behind defining the beginning of motor block as the time between completion of the injection and the ability to move a finger, rather than the total absence of movement in the forearm and hand, as addressed in previous studies [30, 31, 39]. This is advantageous for preserving motor function in patients.

At 20 min, the BIS decreased to 81.9 ± 8.11 and stayed consistently low for 60 min. At 35 min, the BIS was the lowest at 73.05 ± 7.8, and participants were comfortable, lucid, and easily arousable. Moreover, the sedative effects of DEX are induced primarily through α2-adrenoceptors, instead of gamma-aminobutyric acid receptors, resulting in natural sleep with no depression of respiration [28, 42]. This indicates that perineural administration of DEX produces an appropriate sedative effect that is distinct from the systemic route. The observed reductions in DAP, SAP, and HR, post administration of DEX, were considered the consequence of sedation and effects on cardiovascular activity mediated by α2-adrenoceptors. A limitation of this study was that it did not monitor BIS beyond the 60-minute time point.

The exact mechanism by which DEX enhances UGA BPB remains unclear. Two possible mechanisms are proposed: first, DEX may act not only on α2-adrenergic receptors, but also on nerves through cation currents resulting from hyperpolarization [26, 40]. Alternatively, DEX may be absorbed into the blood circulation, reaching α2-adrenergic receptors in the locus coeruleus of the brain to induce sedation [43, 44]. A limitation of this study is the absence of a comparison group who received intravenous infusion of DEX.

Although the Holmann and Bromage scores are widely recognized as standard tools, they have only been validated for general anesthesia and lower-limb blocks, and their applicability to upper-extremity surgery is limited. For sensory and motor block evaluations, we employed a three-point pinprick assessment and three-point functional scale, respectively, which have been widely adopted by investigators in this field [34, 35]. By simplifying the scoring dimensions and focusing on upper extremity functional specificity, the current system ensures reliability while aligning better with the requirements of clinical practical compared to traditional tools.

### Limitations

This study has some limitations. First, the plasma levels of LAs and DEX were not measured, which prevented the establishment of a correlation with the BIS or analgesic effects. Second, different doses of DEX were not compared. As in previous studies, a low dose of DEX was selected to minimize commonly known side effects (hypertension and bradycardia). Moreover, this study did not extend the monitoring of hemodynamic alterations or BIS to a prolonged follow-up. Furthermore, the post-operative VAS was greater than 48 h in the preliminary study; however, data collection was interrupted by patient disturbances during normal nighttime sleep, leading to the abandonment of this data.

## Conclusion

In summary, the combination of low doses of ROP and LIDO substantially accelerated sensory and motor onset times relative to ROP alone. Moreover, the addition of 0.75 µg/kg DEX to the LIDO/ROP mixture in patients undergoing surgeries with UGA BPB prolonged the timings of sensory and motor blocks and the analgesic period without adversely impacting the time of block onset. Moreover, DEX facilitated the attainment of a BIS of 70, thereby inducing an appropriate sedative state without side effects. The combination of ROP, LIDO, and DEX represents an effective approach for providing rapid onset, improved analgesic efficacy, and adequate sedation in BPB, while avoiding undesirable effects (limb or finger paralysis). Thus, this regimen has promising clinical applications.

## Supplementary Information

Supplementary Material 1.

Supplementary Material 2.

## Data Availability

The datasets used and analyzed during the current study are available from the corresponding author upon reasonable request.

## Abbreviations

LIDO: Lidocaine
ROP: Ropivacaine
DEX: Dexmedetomidine
LA: Local anesthetics
BPB: Brachial plexus block
ERAS: Enhanced Recovery After Surgery
SAP: Systolic arterial pressure
DAP: Diastolic arterial pressure
MAP: Mean arterial pressure
BP: Blood pressure
RR: Respiratory rate
BIS: Bispectral index
HR: Heart rate
ANOVA: Repetitive measure analysis of variance
