# Supplementary material for: Evaluation of dexmedetomidine as an adjuvant to low-concentration lidocaine/ropivacaine mixtures in ultrasound-guided axillary brachial plexus block
Source: BMC Anesthesiol. 2025 Jul 17;25:347. doi: 10.1186/s12871-025-03221-9 (PMC12273280; doi:10.1186/s12871-025-03221-9)
Supplement: Supplementary file 2 — Supplementary Material 2. [file 12871_2025_3221_MOESM2_ESM.pdf]

editage

Editing Certificate

This document certifies that the manuscript listed below has been edited to ensure language and grammar accuracy and is error free in these aspects. The edit was performed by professional editors at Editage, a brand of Cactus Communications. The author's core research ideas were not altered in any way during the editing process. The quality of the edit has been guaranteed, with the assumption that our suggested changes have been accepted and the text has not been further altered without the knowledge of our editors.

MANUSCRIPT TITLE

Evaluation of Dexmedetomidine as an Adjuvant to Low-Concentration Lidocaine/Ropivacaine Mixtures in Ultrasound-Guided Axillary Brachial Plexus Block

AUTHORS

Qi Wang

ISSUED ON

April 22, 2025

JOB CODE

EFXIH_2

THE EDITAGE Promise QUALITY GUARANTEE

Prabh Grewal
Senior Vice President - Editage

editage helping you get published

Since 2002, Editage has helped over 430,000 authors publish around 1.2 million research papers in scholarly journals across over 1000 disciplines through editorial, translation, transcription, and publication support services. Editage is a brand of Cactus Communications (cactusglobal.com), a science communication and technology company.

CACTUS.

GLOBAL :
+1(669) 272-1214 | request@editage.com

CHINA :
400-001-8237; 021-60209400 |
fabiao@editage.cn
